# Comparative Analysis of Gene Correlation Networks of Breast Cancer Patients Based on Mutations in TP53

**DOI:** 10.3390/biom12070979

**Published:** 2022-07-13

**Authors:** Byungkyu Park, Jinho Im, Kyungsook Han

**Affiliations:** 1 Department of Bioinformatics, Ichrogene Incorporated, Suwon 16229, Korea; bk.park@ichrogene.com; 2 Department of Computer Engineering, Inha University, Incheon 22212, Korea; jim.go@kakaocorp.com; 3 Data Infomation Platform, Kakao Corporation, Seongnam 13494, Korea

**Keywords:** gene correlation network, prognosis, breast cancer, TP53 mutation

## Abstract

Breast cancer is one of the most prevalent cancers in females, with more than 450,000 deaths each year worldwide. Among the subtypes of breast cancer, basal-like breast cancer, also known as triple-negative breast cancer, shows the lowest survival rate and does not have effective treatments yet. Somatic mutations in the TP53 gene frequently occur across all breast cancer subtypes, but comparative analysis of gene correlations with respect to mutations in TP53 has not been done so far. The primary goal of this study is to identify gene correlations in two groups of breast cancer patients and to derive potential prognostic gene pairs for breast cancer. We partitioned breast cancer patients into two groups: one group with a mutated TP53 gene (mTP53) and the other with a wild-type TP53 gene (wtTP53). For every gene pair, we computed the hazard ratio using the Cox proportional hazard model and constructed gene correlation networks (GCNs) enriched with prognostic information. Our GCN is more informative than typical GCNs in the sense that it indicates the type of correlation between genes, the concordance index, and the prognostic type of a gene. Comparative analysis of correlation patterns and survival time of the two groups revealed several interesting findings. First, we found several new gene pairs with opposite correlations in the two GCNs and the difference in their correlation patterns was the most prominent in the basal-like subtype of breast cancer. Second, we obtained potential prognostic genes for breast cancer patients with a wild-type TP53 gene. From a comparative analysis of GCNs of mTP53 and wtTP53, we found several gene pairs that show significantly different correlation patterns in the basal-like breast cancer subtype and obtained prognostic genes for patients with a wild-type TP53 gene. The GCNs and prognostic genes identified in this study will be informative for the prognosis of survival and for selecting a drug target for breast cancer, in particular for basal-like breast cancer. To the best of our knowledge, this is the first attempt to construct GCNs for breast cancer patients with or without mutations in the TP53 gene and to find prognostic genes accordingly.

## 1. Introduction

Breast cancer is the most common cancer in women worldwide and the second most common cancer overall. More than 1,300,000 patients and 450,000 deaths occur worldwide each year due to breast cancer. The disease is heterogeneous and classified into several subtypes, including luminal, HER2-enriched, and basal-like subtypes, based on the expression of estrogen receptor (ER), progesterone receptor (PR), and the receptor tyrosine kinase ErbB-2 (HER2). The effect of treatments is different in different subtypes, so each subtype of breast cancer requires a different treatment. The HER2-enriched subtype of breast cancer shows the highest survival rate of HER2-targeted treatments [1,2]. In contrast, the basal-like subtype of breast cancer, also known as triple-negative breast cancer (TNBC), shows the lowest survival rate and does not have effective treatments yet [3,4]. Recent studies reported that the TNBC subtype is much more difficult to treat than the other subtypes of breast cancer due to the lack of experimental data [5,6,7] and the interaction between CTL (cytotoxic T lymphocytes) and TNBC cells [8].

Several genes associated with breast cancer risk have been identified. Perhaps the most well-known breast cancer genes are BRCA1 and BRCA2. These genes are involved in DNA repair, and mutations in them are associated with a significant increase in the risk of breast cancer and ovarian cancer. Other genes such as CHEK2 and ATM also function in DNA repair, but their role in breast cancer development is not fully understood.

Many studies for finding prognostic genes for cancer have focused on identifying individual genes that are differentially expressed in cancer cells and normal cells. For example, Gov and Arga [9] identified a prognostic gene module by comparing gene expression levels in epithelial cells from the ovarian tumor and healthy samples. Other studies by Shi et al. [10], Clarke et al. [11], Yang et al. [12], and Paci et al. [13] attempted to find prognostic genes by analyzing network modules in gene correlation networks using the R package WGCNA [14].

The primary focus of this study is to construct gene correlation networks for the prognosis of breast cancer. In an effort to find potential prognostic gene pairs for breast cancer, we partitioned the patients with breast cancer into two groups, one with a wide-type TP53 gene (hereafter called wtTP53) and the other with somatic mutations in the TP53 gene (called mTP53), and constructed gene correlation networks for the two groups. There are several reasons that we selected the TP53 gene for classifying gene expression samples. First, TP53 mutations occur frequently in all breast cancer subtypes. Second, TP53 mutations are known to be associated with poor prognosis in breast cancer [15,16] and other cancers [17].

Our approach is different from the other works [10,11,12] in several ways: (1) We divided breast cancer patients into two groups according to the existence of somatic mutations in TP53, whereas the other works treated breast cancer patients as a whole or divided them into predicted molecular subtypes. (2) They built weighted gene correlation networks using the R package WGCNA, but we did not use the WGCNA package to construct correlation networks. (3) They attempted to find prognostic genes by analyzing network modules found by WGCNA, but we selected genes with significantly different correlations in two groups and performed the log-rank test with different combinations of the genes to find potential prognostic gene pairs.

The rest of this paper presents our approach to constructing GCNs for two groups, the results of a comparative analysis of GCNs, and the best prognostic gene pairs found for the two groups. To the best of our knowledge, this is the first attempt to construct GCNs for breast cancer patients with or without mutations in the TP53 gene and to obtain prognostic gene pairs accordingly. One important finding of our study is that the prognostic power of a gene pair can be substantially different depending on the existence of a mutant TP53 gene and that a gene pair is better than a single gene in predicting the survival time of patients.

## 2. Materials and Methods

**Figure 1:**
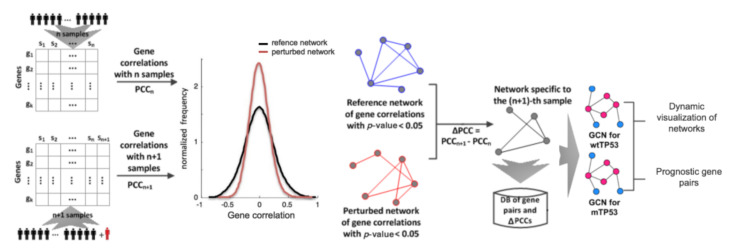
Schematic overview of the framework for constructing gene correlation networks (GCNs) and obtaining prognostic gene pairs for two groups of breast cancer patients. wtTP53: a group of breast cancer patients with a wild-type TP53 gene. mTP53: a group of breast cancer patients with somatic mutations in the TP53 gene.

This section discusses our approach to constructing gene correlation networks (GCNs) and obtaining prognostic gene pairs for breast cancer patients. The schematic overview of our approach is shown in Figure 1.

### 2.1. Gene Expression Data of Breast Cancer Patients and Grouping of Patients

We obtained gene expression data of the primary tumor samples of 1050 breast cancer patients in the TCGA-BRCA project [18] from the NIH GDC Data Portal (https://portal.gdc.cancer.gov/repository accessed on 2 December 2019). The number of patients tested for somatic mutations was 750, so the remaining 300 patients were excluded. As a result, a total of 750 TCGA-BRCA patients were analyzed in our study (Supplementary Material S1).

Table 1 shows the number of breast cancer patients and their subtypes based on the PAM50 gene expression assay [19]. A cancer is called estrogen-receptor-positive (or ER+) if it has receptors for estrogen. Similarly, a cancer is called progesterone receptor-positive (PR+) if it has receptors for progesterone. Among the breast cancer subtypes, the normal-like, luminal A, luminal B, and HER2-enriched subtypes have favorable clinical outcomes. The majority of luminal A is ER+/PR+ and HER2-, whereas around 30% of luminal-B are ER+/PR+ and HER2+ [20,21]. On the other hand, the basal-like subtype is hormone receptor-negative (ER-, PR-, and HER2-) and is also called triple-negative breast cancer (TNBC).

Regardless of the subtypes, we partitioned the 750 breast cancer patients into two groups: a group of 496 patients with a wild-type TP53 gene (called wtTP53 in this paper) and another group of 254 patients with somatic mutations in the TP53 gene (called mTP53). The reason that we divided the patients into two groups with respect to the existence of somatic mutations in the TP53 gene is that TP53 mutations occur in all breast cancer subtypes and TP53 mutations are known to be associated with poor prognosis in breast cancer [15,16].

### 2.2. Selecting Core Genes

We obtained a total of 516 genes associated with BRCA (74 elite and 442 related genes) from MalaCards database [22]. What we call core genes are comprised of the elite genes of MalaCards and the genes that are potentially associated with breast cancer. We first obtained 74 elite genes of breast cancer from MalaCards, which are defined as those with strong evidence for association with the disease. For comparing core genes and PAM50 [19], we selected an additional 50 genes of PAM50.

### 2.3. The Prognostic Power of Core Genes

According to the Genomic Data Commons (GDC) (https://gdc.cancer.gov/clinical-data-elements accessed 2 December 2019), the TCGA clinical data can have three properties for survival analysis:

1. days_to_last_follow_up: time interval from the date of initial pathologic diagnosis to the date of the last follow-up, represented as a calculated number of days
2. days_to_death: the number of days from the date of the initial pathologic diagnosis to the date of death for the case in the investigation
3. vital_status: the state of being living or deceased for cases that are part of the investigation

In the above three properties, we used the first two properties (days_to_last_follow_up and the days_to_death), which correspond to overall survival. The overall survival time represents the time interval from the date of initial pathologic diagnosis to the date of either death or last follow-up.

We evaluated the prognostic power of core genes by the Cox proportional hazard model [23]. For each pair of core genes in wtTP53 and mTP53, we computed a gene pair of the hazard ratio of the Cox model in the data.

### 2.4. Constructing Gene Correlation Networks

For every pair of core genes in wtTP53 and mTP53, we computed the Pearson correlation coefficients (PCC) between their expression levels by Equation (1). In the equation, *N* is the number of patients and $\bar{x}$ is the mean of *x*.

(1) $$PCC\left(x_{i},x_{j}\right)=\frac{\sum_{k=1}^{N}\left(x_{ik}-{\bar{x}}_{i}\right)\left(x_{jk}-{\bar{x}}_{j}\right)}{\sqrt{\sum_{k=1}^{N}{\left(x_{ik}-{\bar{x}}_{i}\right)}^{2}\sum_{k=1}^{N}{\left(x_{jk}-{\bar{x}}_{j}\right)}^{2}}}$$

We then constructed gene correlation networks (GCNs) for wtTP53 and mTP53. In GCNs, PCCs were used as the weights of the edges between genes. From the comparative analysis of GCNs for wtTP53 and mTP53, we selected gene pairs that satisfy three criteria: (1) PCCs of the gene pair have the *p*-value of PCC <0.05 in n normal samples of GTEx breast, (2) we added a single sample of the patient to the n normal samples and selected those gene pairs with a *p*-value of PCC <0.05 after recomputing PCC with n+1 samples, (3) PCCs of the gene pair from two GCNs (*n* normal samples and n+1 samples) should be significantly different between the normal samples and the patient sample by Equation (2).

(2) $$\Delta PCC\left(x_{i},x_{j}\right)=\left|PCC_{n+1}\left(x_{i},x_{j}\right)-PCC_{n}\left(x_{i},x_{j}\right)\right|$$

### 2.5. Finding Prognostic Genes for Two Groups of Patients

We performed the log-rank test [24] separately for the two groups. In each of the wtTP53 and mTP53 groups, we clustered the patients further into two clusters by hierarchical clustering and performed the log-rank test for gene pairs of core genes with the *p*-value of PCC <0.05 to examine the difference in their survival time and obtained the *p*-value of the test. It should be noted that patients can be clustered differently depending on the gene pairs used for clustering. For each group, we selected the gene pairs which has the lowest *p*-value in the log-rank test. The *p*-value was adjusted using the Benjamini–Hochberg procedure, which consists of the following steps to control the false discovery rate at level α.

1. Order the *p*-values as $p_{1},p_{2},\ldots ,p_{m}$.
2. Find the rank *j* for which $p_{j}\leq \frac{j}{m}\alpha$.
3. Declare the top *j* tests 1, 2, …, *j* as significant.

$p_{j}\leq \frac{j}{m}\alpha$ in the second step can be transformed to $p_{j}\frac{m}{j}\leq \alpha$, so $min(1,p_{j}\frac{m}{j})$ was used as an adjusted *p*-value.

To find the gene pairs for prognosis of survival time of the wtTP53 and mTP53 groups, we computed n(k2)ΔPCCs for k core genes in n tumor samples.

## 3. Results

### 3.1. Prognostic Genes and Gen Pairs for Two Patient Groups

**Figure 2:**
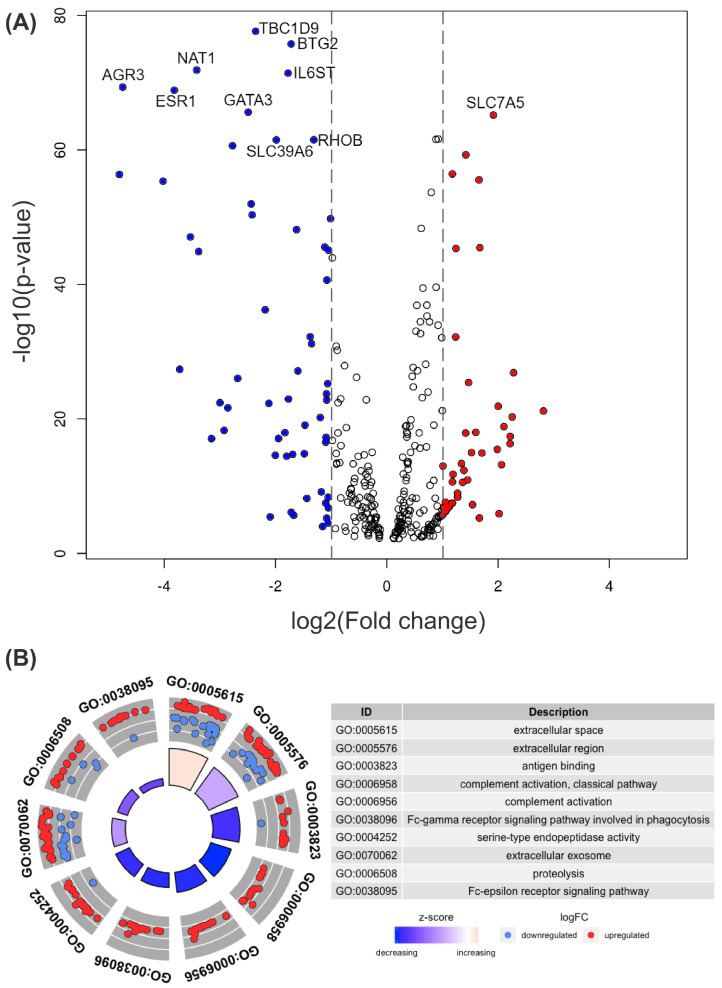
(**A**) Volcano plot for comparing differentially expressed genes in two groups, wtTP53 and mTP3. The horizontal axis represents the fold change (FC) between the two groups on a log2 scale, and the vertical axis shows the negative logarithm to the base 10 of *p* values from the *t*-test. A gene with a higher expression level in mTP53 than in wtTP53 has a positive FC and is shown as a red dot. A gene with a lower expression level in mTP53 than in wtTP53 has a negative FC and is shown as a blue dot. Top 10 genes with low adjusted *p*-values by Benjamini–Hochberg procedure are labeled with their names. (**B**) GO circle plot and GO terms for genes with significant FC. Genes with higher expression levels in mTP53 than in wtTP53 are shown as red dots, and genes with lower expression levels in mTP53 are shown as blue dots. The z-score is the number of overexpressed genes minus the number of underexpressed genes divided by the square root of the count.

There were 105 differentially expressed genes more than twice in one group than the other with an adjusted *p*-value < 0.05. Figure 2A shows a volcano plot of differentially expressed genes in two groups, wtTP53 and mTP3. Among the 516 core genes, 321 genes with an adjusted *p*-value <0.01 are shown in the volcano plot (Supplementary Material S2).

Differentially expressed genes [25] with significant fold change (FC) between wtTP53 and mTP3 were selected and tagged with GO [26,27] terms (Supplementary Material S3) in Figure 2B.

To find prognostic gene pairs for the wtTP53 and mTP53 groups, we computed 496·(5162)= 65,903,520 ΔPCCs for wtTP53, 261·(5162)= 34,679,070 ΔPCCs for mTP53 (see Table 1 for the number of tumor samples). PCCs from the 100 normal samples and 100+1 samples groups were compared using the R package cocor [28]. We selected only those gene pairs with ΔPCC from the normal samples greater than the average of ΔPCCs of gene pairs. There were a total of 46,878 distinct gene pairs in two groups of wtTP53 and mTP53. The gene pairs are available in Supplementary Material S4.

**Figure 3:**
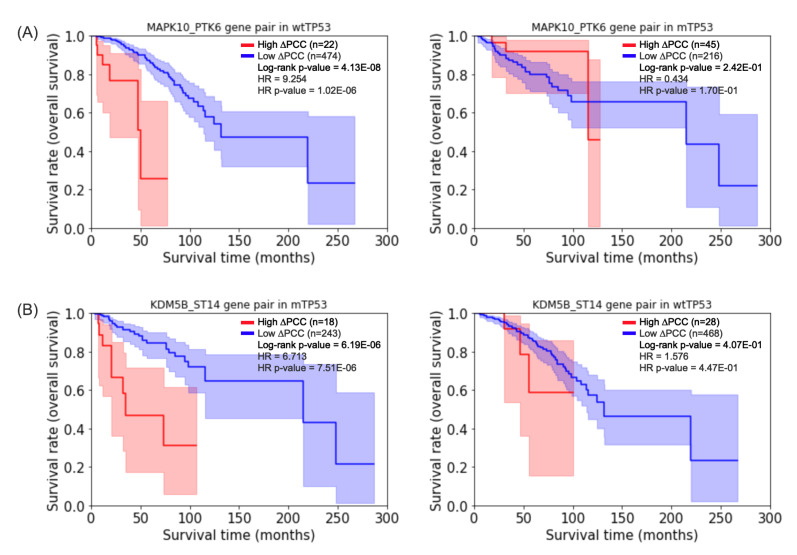
Kaplan–Meier plots for the gene pairs selected by the log-rank test [24]. (**A**) The highest hazard ratio of gene pair MAPK10_PTK6 for prognosis of survival of wtTP53 (left plot). (**B**) The highest hazard ratio of gene pair KDM5B_ST14 for prognosis of survival of mTP53 (**left plot**). The Kaplan–Meier plots of the same gene pairs are displayed for the other group (**right plots**) for comparative purposes.

Table 2 shows 10 gene pairs with the highest hazard ratios in patients with wtTP53, and Table 3 shows 9 gene pairs with the highest hazard ratios in patients with mTP53. Figure 3 shows the Kaplan–Meier plots [29] of prognostic gene pairs for wtTP53 and mTP53. For comparative purposes, the Kaplan–Meier plots of the same prognostic genes are displayed for mTP53 or wtTP53 as well. For example, an adjusted *p*-value [30] of the log-rank test of the gene pair MAPK10_PTK6 <0.05 in wtTP53 (the left plot of Figure 3A) but an adjusted *p*-value of the log-rank test of the gene pair >0.05 in mTP53 (the right plot of Figure 3A). On the other hand, an adjusted *p*-value of the log-rank test of the gene pair KDM5B_ST14 <0.05 in mTP53 (the left plot of Figure 3B) but an adjusted *p*-value of the log-rank test of the gene pair >0.05 in wtTP53 (the right plot of Figure 3B).

The results of the prognostic analysis suggest that prognostic gene pairs for breast cancer can be significantly different depending on the existence of a mutant TP53 gene. The log-rank test and the Cox PH model of the gene pairs are available in Supplementary Material S5.

### 3.2. A Comparison of TCGA-BRCA and an Independent Validation Cohort

For the independent validation cohort, we obtained gene expression data (Dataset ID: EGAD00010000210) of 997 breast cancer patients in the METABRIC [31] from the European Genome-phenome Archive (EGA). There are 721 breast cancer patients with wtTP53, 99 patients with mTP53, and 177 patients having no information about the mutation type of the TP53 gene. There are seventeen patients in wtTP53 without the survival time, so we selected 704 patients in wtTP53 of METABRIC.

**Figure 4:**
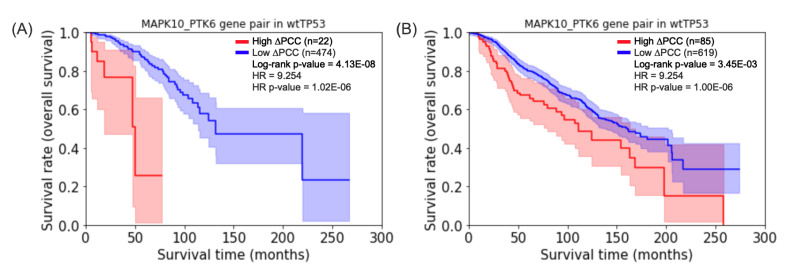
Kaplan–Meier plots for the gene pair MAPK10_PTK6. (**A**) The gene pair for prognosis of survival of wtTP53 in TCGA-BRCA. (**B**) The gene pair for prognosis of survival of wtTP53 in METABRIC.

For comparative purposes, we computed 704·(5162)= 93,540,480 ΔPCCs for wtTP53 in the METABRIC, 99·(5162)= 13,154,130 ΔPCCs for mTP53 in the METABRIC. Figure 4 shows that the highest hazard ratio of gene pair MAPK10_PTK6 for the prognosis of survival of wtTP5 in TCGA-BRCA is shown a similar prognosis of survival of mTP53 in METABRIC. On the other hand, the highest hazard ratio of gene pair KDM5B_ST14 for mTP53 in TCGA-BRCA has no significant *p*-value for the log-rank test for the prognosis of survival of mTP53 in METABRIC. The log-rank test and the Cox PH model of the gene pairs of METABRIC are available in Supplementary Material S6.

### 3.3. Comparison of Gene Correlations with Gene Expressions in Classifying Subtypes of Breast Cancer

Classification of subtypes of breast cancer is not the focus of our study, but we investigated whether PCCs and ΔPCCs of gene pairs are useful in classifying subtypes of breast cancer as well. We constructed a 4-class (basal-like, HER2-enriched, luminal A, and luminal B) classifier with linear regression in Keras (https://keras.io accessed on 2 December 2019) on TensorFlow (https://www.tensorflow.org accessed on 2 December 2019). The classifier consists of 9 layers (including input and output layers). Default values were used for parameters β1 and β2 in the Adam optimizer (https://keras.io/api/optimizers/adam accessed on 2 December 2019). For classification, we additionally computed 757·(502)= 927,325 ΔPCCs (among 757 ΔPCCs, 496 are for wtTP53 and 261 are for mTP53). 70% of the dataset was used for training the classifier, and the remaining 30% was used for testing the classifier.

For comparative purposes, we constructed two classification models which use different features. As for features, one model used PCCs and ΔPCCs of gene pairs of 50 genes in PAM50. The other model used gene expression levels of 50 genes in PAM50 for features. As shown in Table 4, the model using PCCs and ΔPCCs of gene pairs exhibited better performance than the model using gene expressions of genes in all subtypes. In particular, the model with PCCs and ΔPCCs was consistently better than the other model in F-score and MCC. These results indicate that PCCs and ΔPCCs of gene pairs can be useful in classifying subtypes of breast cancer as well.

## 4. Discussion

Breast cancer is one of the most heterogeneous cancers with many subtypes, and different subtypes require different treatments. A subtype such as basal-like breast cancer, also known as triple-negative breast cancer (TNBC), shows the lowest survival rate and does not have targeted treatments yet. Due to the lack of biomarkers that can distinguish TNBC from other subtypes and definitive targets, there are few therapeutic interventions for TNBC [32] and chemotherapy has been the primary treatment in the past decades [33].

In an effort to find potential prognostic biomarkers for breast cancer, we analyzed gene expression data of 750 breast cancer patients. For the two groups of patients, we constructed gene correlation networks (GCNs) enriched with prognostic information. Our GCN is more informative than typical GCNs in the sense that it indicates the type of correlation between genes, the concordance index, and the prognostic type of a gene (Supplementary Material S2).

**Figure 5:**
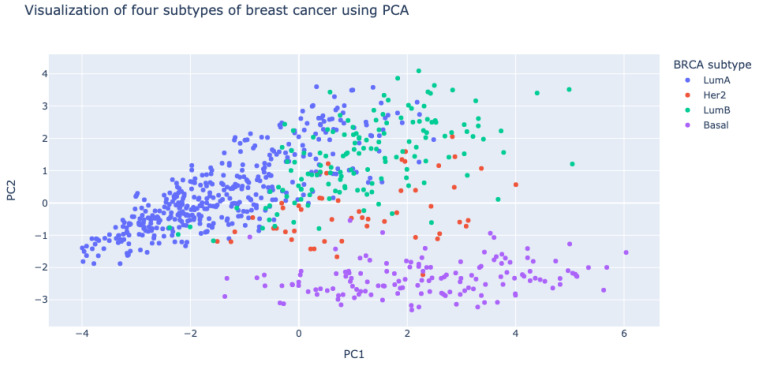
Visualization of four subtypes of breast cancer with respect to ΔPCCs of gene pairs. Principal component analysis (PCA) was used for the visualization. ΔPCCs of gene pairs in the basal-like subtype (violet dots) are very different from those in the other subtypes.

We performed principal component analysis (PCA) of ΔPCCs of gene pairs in 750 breast cancer patients using the scikit-learn package (https://scikit-learn.org/ accessed on 2 December 2019). It is interesting to note that ΔPCCs of gene pairs in the basal-like subtype (shown in purple dots in Figure 5) are very different from those in the other subtypes (luminal A, luminal B, and HER2-enriched subtypes) of breast cancer.

The R package WGCNA is perhaps the most widely used software for analyzing inter-gene correlations from gene expression data. With the same gene expression data of 455 core genes used in our work, we analyzed gene correlation and constructed a gene correlation network using WGCNA. As shown in Supplementary Material S7, four modules were found by WGCNA, and the turquoise color module contains many gene pairs specific to wtTP53. No modules specific to mTP53 were found by WGCNA. A comparison of our GCN for wtTP53 and the GCN found by WGCNA shows that the two GCNs have 94 genes and 77 gene pairs in common. However, more than 59% (136 out of 230) of the total genes and 95% (1673 out of 1750) of the total gene pairs are found in one GCN only. The 108 genes and 489 gene pairs in our GCN could not be found by WGCNA. WGCNA is useful for automatically finding modules from gene expression data and analyzing them but is not convenient for comparing user-specified groups. As mentioned earlier, a comparison of the patients with wtTP53 to the patients with mTP53 cannot be made using WGCNA because WGCNA cannot find a module specific to wtTP53 or mTP53.

To examine the sensitivity of potential prognostic gene pairs to possible outliers in gene expressions, we randomly removed 10% of breast cancer samples and performed the survival analysis with the remaining 90% of the samples. Due to the randomness of selected samples, the survival analysis was repeated three times in each of the log-rank test and Cox proportional hazard (Cox PH) models. In patients with wtTP53, eight potential prognostic gene pairs out of the ten prognostic gene pairs shown in Table 2 remained significant. In the log-rank test with 90% of patients with mTP53, there were no gene pairs with the adjusted *p*-value (FDR) < 0.05. However, in the Cox proportional hazard model, five gene pairs out of the nine prognostic gene pairs (Table 3) were significant (that is, their *p*-values of the Cox PH model < 0.0001). One reason for finding fewer potential prognostic gene pairs in 90% of the patients with mTP53 than in 90% of the patients with wtTP53 seems to be the smaller number of patients with mTP53. Initially, the number of patients with wtTP53 was 496, whereas the number of patients with mTP53 was 254, which is about half of the patients with wtTP53. With more patients available for the survival analysis, we expect the potential prognostic gene pairs to be more stable and less sensitive to outliers. Details are available in Supplementary Material S8.

We also tried Spearman’s rank correlation coefficient (SCC) to find potential prognostic gene pairs. Ten potential prognostic gene pairs derived with SCC in patients with wtTP53 are given in Supplementary Material S9. Among the ten potential prognostic gene pairs, AKIP1_SUSD2 is the only one common to the potential prognostic gene pairs derived with PCC (Supplementary Material S5). In patients with mTP53, 16 gene pairs were found as potential prognostic gene pairs from the survival analysis with SCC. There were no prognostic gene pairs common to the 16 gene pairs derived with SCC and the nine prognostic gene pairs derived with PCC (Table 3). These results indicate that potential prognostic gene pairs can be changed depending on the types of gene correlations, but the way of deriving potential prognostic gene pairs still works with different types of correlations.

The main contribution of our work is that we performed a comparative analysis of two groups of breast cancer patients based on mutations in the TP53 gene and constructed gene correlation networks for the two groups. We also derived potential prognostic gene pairs from the networks and observed that the two groups do not share potential prognostic genes. As for another contribution of our work, we discovered that the prognosis of breast cancer patients is significantly different depending on the existence of the mutant TP53 gene and that gene pairs are more prognostic of survival than single genes in patients with a wild-type TP53 gene.

Although the results of our approach are promising in finding potential prognostic gene pairs in breast cancer, there are a few limitations. First, potential prognostic gene pairs can be changed depending on the types of correlations used for deriving gene pairs. Second, potential prognostic gene pairs may be sensitive to outliers in gene expressions when there are not enough samples.

## 5. Conclusions

In this study, we partitioned the 750 breast cancer patients into two groups, one with a wild-type TP53 gene (wtTP53) and the other with somatic mutations in the TP53 gene (mTP53), and constructed gene correlation networks (GCNs) enriched with prognostic information. Comparative analysis of the two GCNs revealed several interesting findings. First, many genes show different expression levels in the two patient groups. Second, but more importantly, there are several gene pairs in the two GCNs and their difference in correlation patterns is the most prominent in the basal-like subtype of breast cancer.

From the survival analysis, we identified three prognostic gene pairs {MAPK10_PTK6, ECT2_HIF1A-AS2, HIF1A-AS2_KIF15} for the wtTP53 group, and another three prognostic genes {KDM5B_ST14, NAT2_PBOV1, KIT_RHOBTB2} for the mTP53 group. These results suggest that the prognosis of breast cancer patients is significantly different depending on the existence of the mutant TP53 gene and that gene pairs are more prognostic of survival than single genes in patients with a wild-type TP53 gene.

The enriched GCNs and prognostic gene pairs identified in this study will be informative for the prognosis of survival and for selecting a drug target for breast cancer, in particular for the basal-like subtype of breast cancer. The GCNs and data are available at http://bclab.inha.ac.kr/brca accessed on 2 December 2019.

## Figures and Tables

**Table 1:** The number and proportion (in parentheses) of breast cancer cases from TCGA and their subtypes based on the PAM50 classification [19]. The proportion represents the ratio of the cases to the entire cases of the same subtype. mTP53: breast cancer patients with somatic mutations in the TP53 gene. wtTP53: breast cancer patients with a wild-type TP53 gene.

Breast Cancer Subtype	mTP53	wtTP53	Total
Luminal A	41 (16.1%)	369 (74.4%)	410 (54.7%)
Luminal B	50 (19.7%)	95 (19.2%)	145 (19.3%)
HER2-enriched	38 (15.0%)	14 (2.8%)	52 (6.9%)
Basal-like	125 (49.2%)	18 (3.6%)	143 (19.1%)
Total	254 (33.9%)	496 (66.1%)	750 (100.0%)

**Table 2:** Gene pairs with the highest hazard ratio in wtTP53.

Gene Pair	Large	Small	Log-Rank Test		Cox PH	
	ΔPCC	ΔPCC	*p*-value	adj. *p*-value	Hazard Ratio	*p*-value
MAPK10_PTK6	22	474	4.13E−08	1.80E−04	9.254	1.02E−06
ECT2_HIF1A-AS2	15	481	2.11E−05	1.48E−02	8.616	9.47E−05
HIF1A-AS2_KIF15	16	480	2.11E−05	1.48E−02	8.616	9.47E−05
CLDN7_MAPK10	16	480	1.75E−06	3.31E−03	8.611	1.02E−05
CDH3_FGFR2	13	483	3.88E−05	2.18E−02	7.950	9.13E−05
PTGS2_SUSD2	20	476	8.62E−09	5.36E−05	7.695	5.77E−06
LSINCT5_SUSD2	14	482	1.01E−05	9.59E−03	6.786	6.11E−05
AHR_SUSD2	29	467	5.06E−10	1.10E−05	6.557	7.85E−07
GLI1_RMST	14	482	1.51E−06	3.14E−03	6.059	3.60E−05
CDH3_GLI1	32	464	1.38E−09	1.88E−05	5.945	1.72E−07

**Table 3:** Gene pairs with the highest hazard ratio in mTP53.

Gene Pair	Large	Small	Log-Rank Test		Cox PH	
	ΔPCC	ΔPCC	*p*-value	adj. *p*-value	Hazard Ratio	*p*-value
KDM5B_ST14	18	243	6.19E−06	4.07E−02	6.713	7.51E−06
NAT2_PBOV1	27	234	7.69E−06	4.07E−02	5.868	3.49E−05
KIT_RHOBTB2	24	237	5.24E−06	4.07E−02	5.703	1.09E−05
PBOV1_TWIST1	49	212	2.80E−07	1.21E−02	5.680	1.51E−06
FLT1_MDM2	30	231	5.81E−07	1.25E−02	5.247	7.97E−06
PIK3CA_PRLR	33	228	1.13E−06	1.63E−02	5.139	6.96E−06
EPCAM_SERPINE1	28	233	6.46E−06	4.07E−02	4.714	1.85E−05
CDC27_MDM2	25	236	6.92E−06	4.07E−02	4.644	9.70E−05
CLDN7_PIK3CA	38	223	8.50E−06	4.07E−02	4.082	6.79E−05

**Table 4:** Comparison of two models with different features in classifying subtypes of breast cancer. A better performance is shown in bold. AC: accuracy, SE: sensitivity, SP: specificity, MCC: Matthews Correlation Coefficient.

Feature	Subtype	AC	SE	SP	PPV	NPV	F-Score	MCC
	Basal-like	**99.71**	**98.08**	100.0	100.00	**99.66**	**0.990**	**0.989**
PCCs &	HER2-enriched	**98.54**	**95.65**	**98.75**	**84.62**	**99.68**	**0.898**	**0.892**
ΔPCCs	Luminal A	**95.32**	94.48	**96.27**	**96.61**	93.94	**0.955**	**0.906**
	Luminal B	**95.91**	**90.32**	97.14	87.50	**97.84**	**0.889**	**0.864**
	Basal-like	99.10	96.15	100.00	100.00	98.84	0.980	0.975
gene	HER2-enriched	96.40	93.33	96.88	82.35	98.94	0.875	0.856
expressions	Luminal A	94.59	**97.83**	92.31	90.00	**98.36**	0.938	0.892
	Luminal B	91.89	70.83	**97.70**	**89.47**	92.39	0.791	0.749

## Data Availability

All appendices and GCNs are available at http://bclab.inha.ac.kr/brca accessed on 2 December 2019.

## Supplementary Materials

The following supporting information can be downloaded at: https://www.mdpi.com/article/10.3390/biom12070979/s1, Supplementary Material S1: 750 Breast Cancer Patients and Their Subtypes; Supplementary Material S2: Concordance Indexes and Fold Changes; Supplementary Material S3: Gene Ontology Annotations Obtained from DAVID; Supplementary Material S4: Gene Pairs in the wtTP53 and mTP53 Groups; Supplementary Material S5: Result of the Log-Rank Test and the Cox PH Model of Gene Pairs in the wtTP53 and mTP53 Groups; Supplementary Material S6: Result of the Log-Rank Test and the Cox PH Model of Gene Pairs in the wtTP53 Group of METABRIC; Supplementary Material S7: The GCN for a Module Found by the R Package WGCNA from Core Genes; Supplementary Material S8: Result of the Log-Rank Test and the Cox PH Model of Gene Pairs Derived from 90% of Samples; Supplementary Material S9: Result of the Log-Rank Test and the Cox PH Model of Gene Pairs Derived by SCC.

## Abbreviations

The following abbreviations are used in this manuscript:

GCNs	Gene correlation networks
wtTP53	a wild-type TP53 gene
mTP53	a mutated TP53 gene
LumA	Luminal A breast cancer
LumB	Luminal B breast cancer
Her2	HER2-enriched breast cancer
Basal	Basal-like or Triple-negative breast cancer
