# Supplementary material for: Comparative Analysis of Gene Correlation Networks of Breast Cancer Patients Based on Mutations in TP53
Source: Biomolecules. 2022 Jul 13;12(7):979. doi: 10.3390/biom12070979 (PMC9313229; doi:10.3390/biom12070979)
Supplement: Supplementary file 1 [file biomolecules-12-00979-s001.zip › Supplementary Materials S7.pdf]

Additional file 6

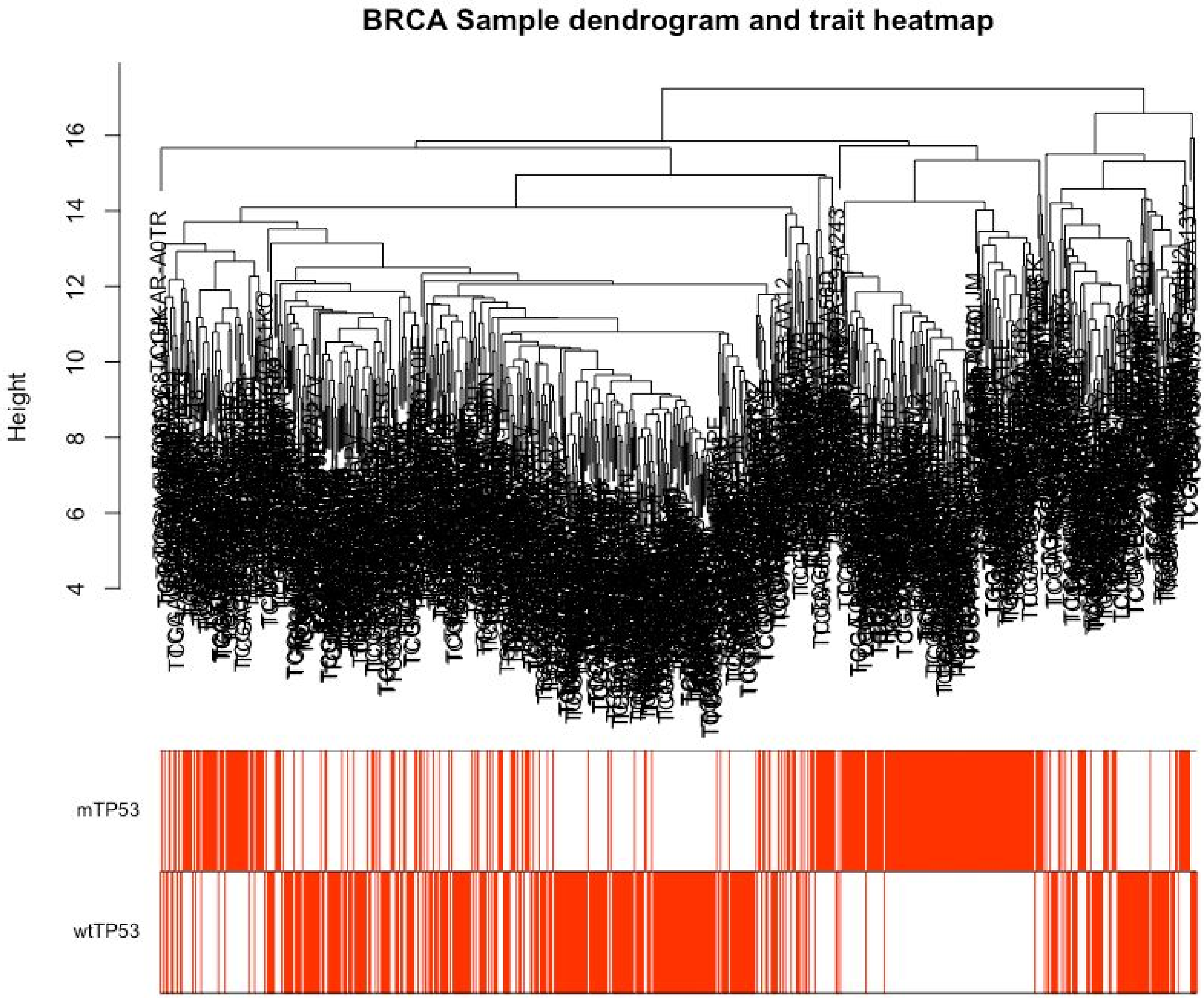

1. Clustering dendrogram of RNA_seq samples based on their Euclidean distance.

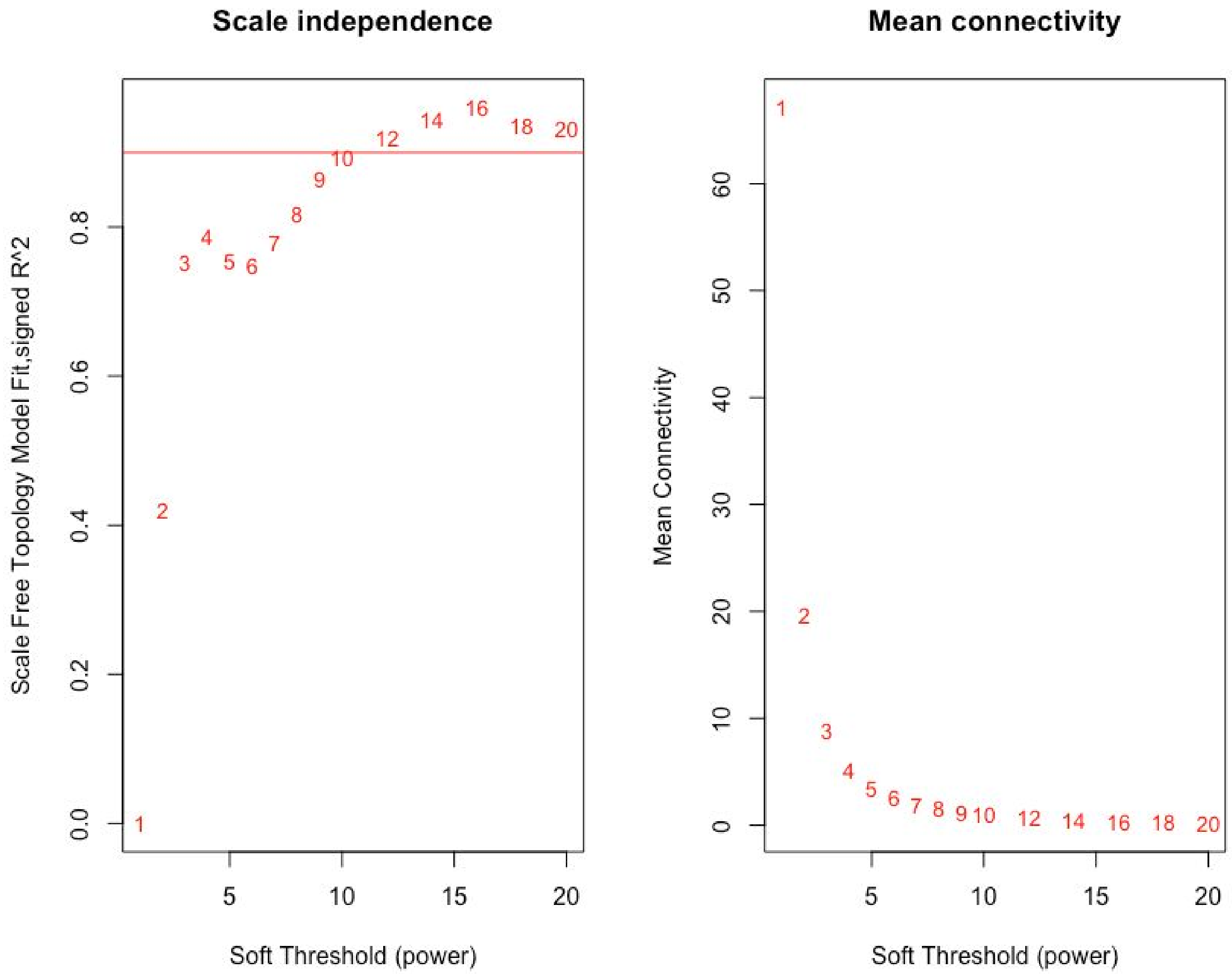

2. Analysis of network topology for various soft-thresholding powers. The left panel shows the scale-free fit index (y-axis) as a function of the soft-thresholding power (x-axis). The right displays the mean connectivity (degree, y-axis) as a function of the soft-thresholding power (x-axis).

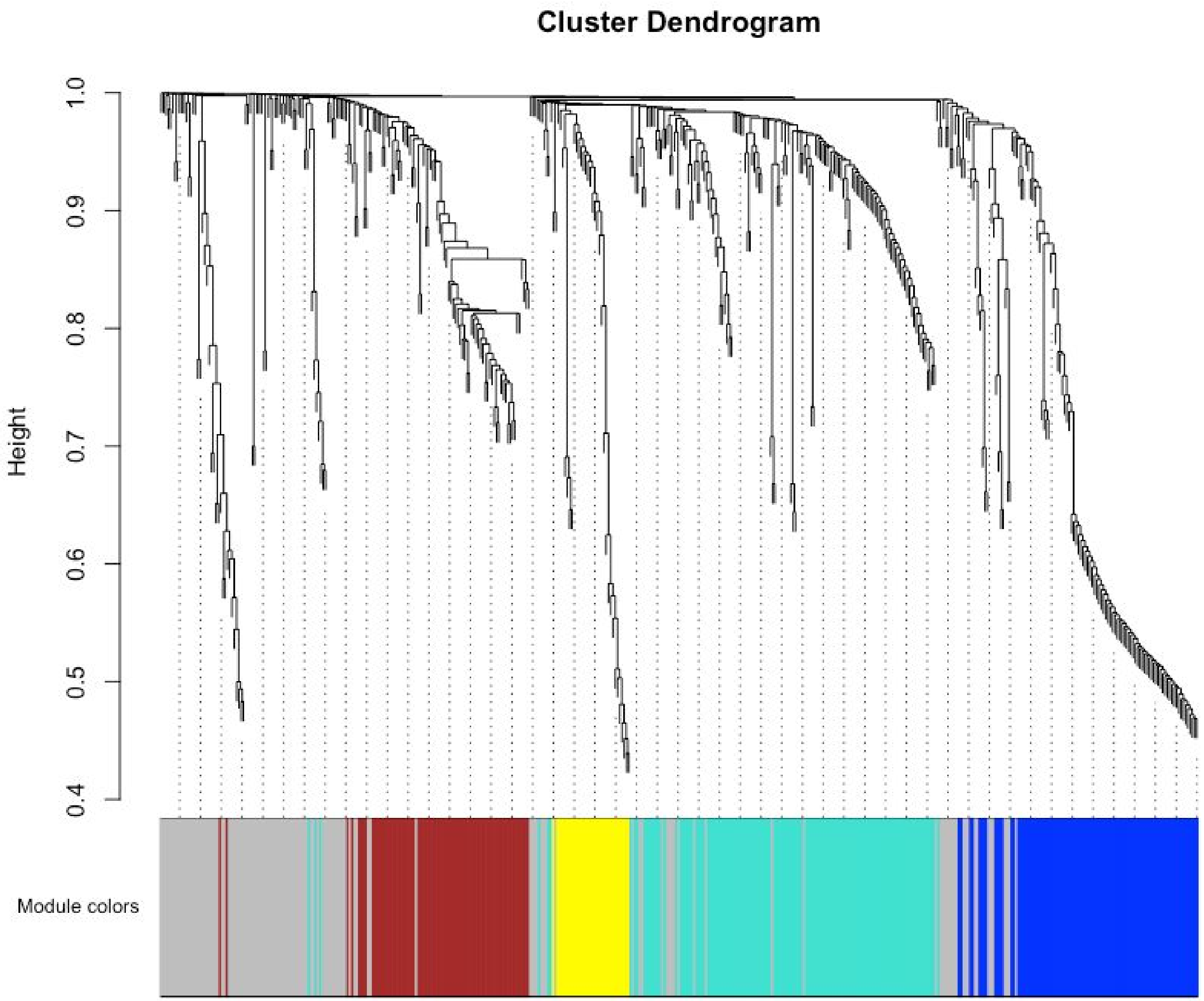

3. Clustering dendrogram of genes, with dissimilarity based on topological overlap, together with assigned module colors.

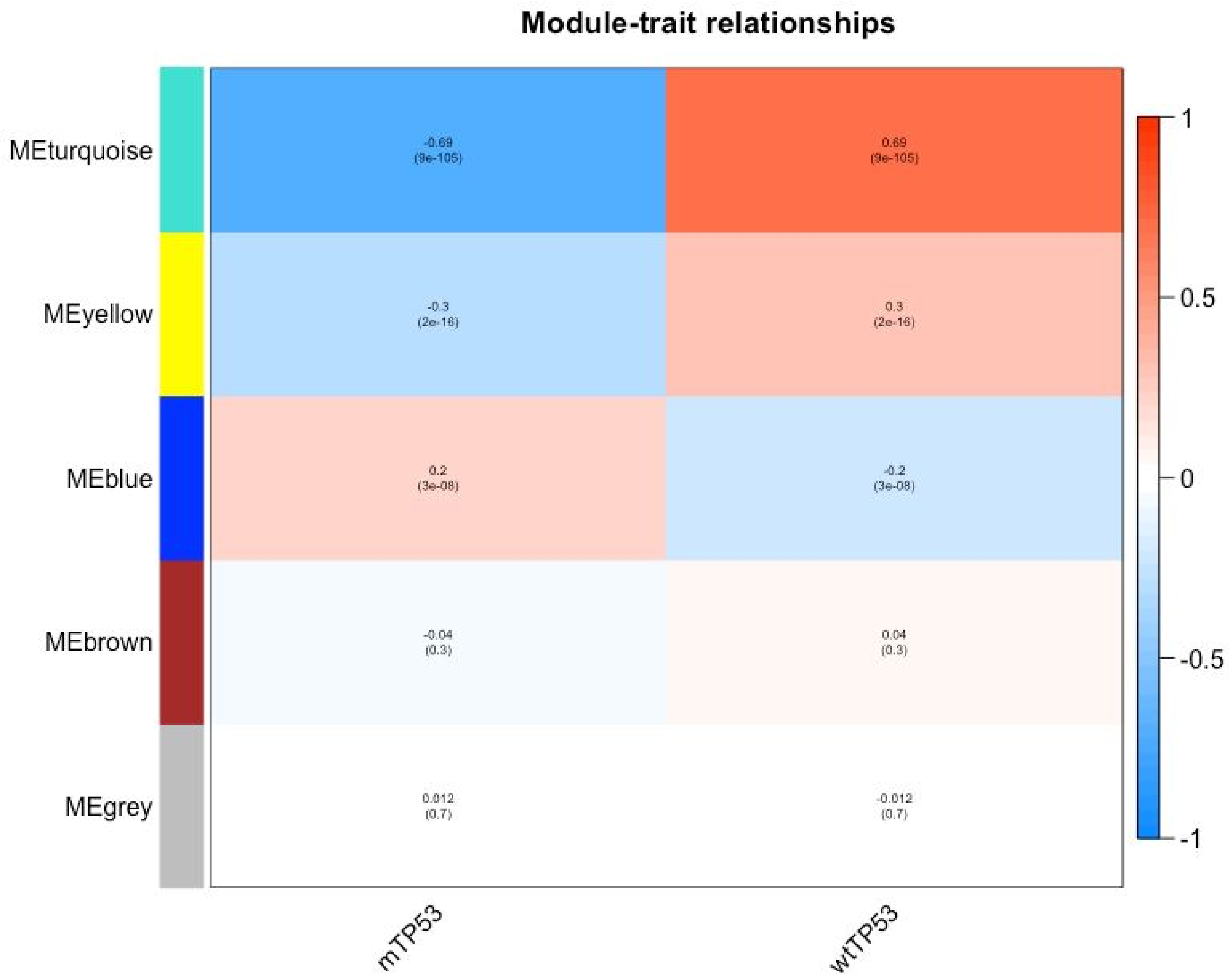

4. Module-trait associations. Each row corresponds to a module eigengene, column to a trait. Each cell contains the corresponding correlation and p-value. The table is color-coded by correlation according to the color legend.

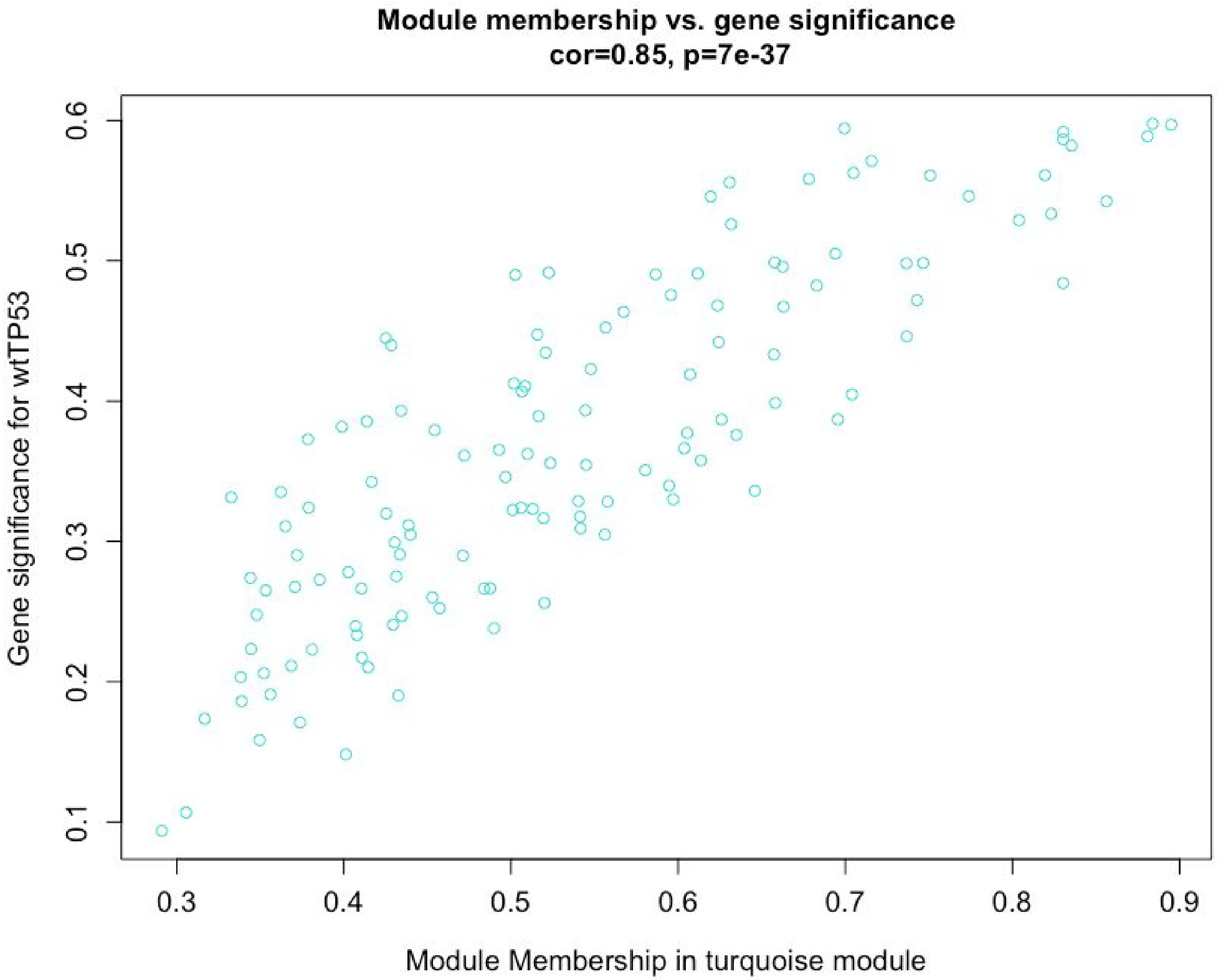

5. A scatterplot of Gene Significance (GS) for wtTP53 vs. Module Membership (MM) in the turquoise module. There is a highly significant correlation between GS and MM in this module.

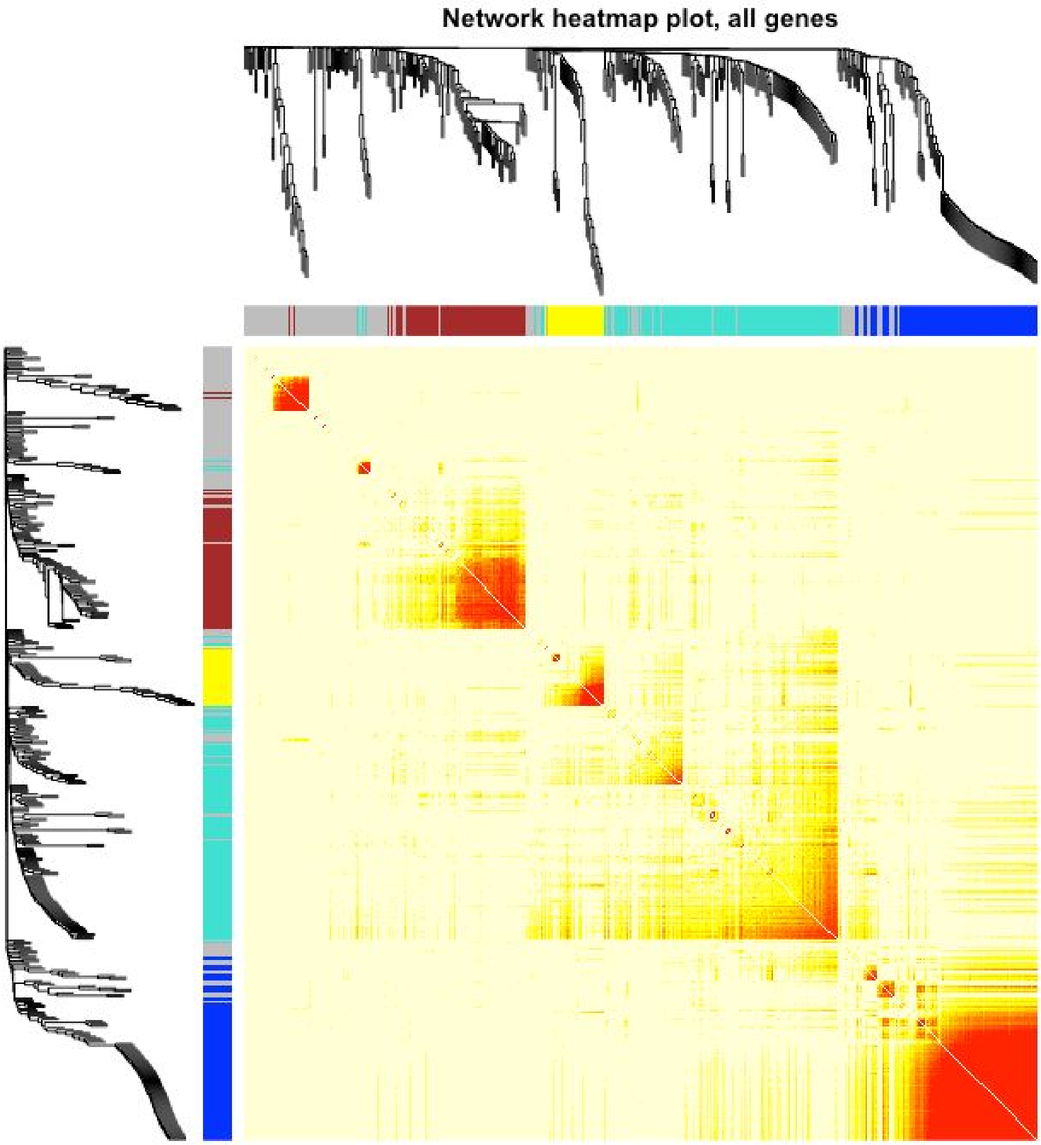

6. Visualizing the gene network using a heatmap plot. The heatmap depicts the Topological Overlap Matrix (TOM) among 455 core genes in the analysis. Light color represents low overlap and progressively darker red color represents higher overlap. Blocks of darker colors along the diagonal are the modules. The gene dendrogram and module assignment are also shown along the left side and the top.

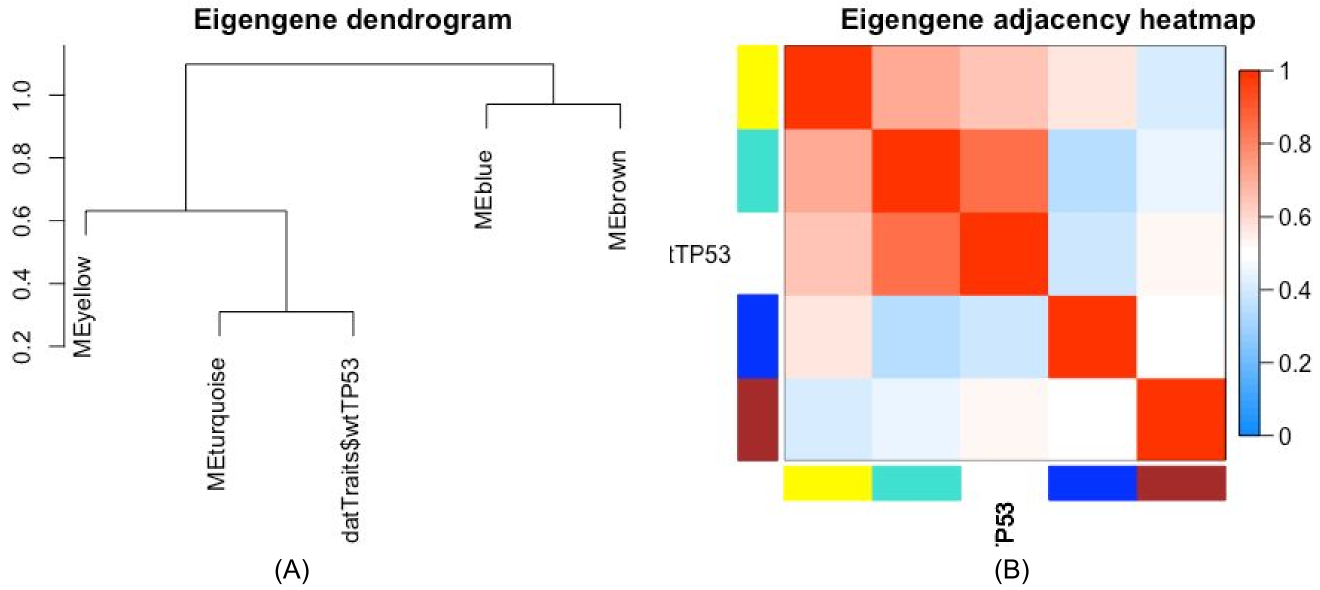

7. Visualization of the eigengene network representing the relationships among the modules and the wtTP53. Panel (A) shows a hierarchical clustering dendrogram of the eigengenes in which the dissimilarity of eigengenes E_I, E_J is given by $1 - cor(E_I, E_J)$. The heatmap in panel (B) shows the eigengene adjacency $A_{IJ} = (1 + cor(E_I, E_J))/2$.
